# Isolated peritoneal carcinomatosis in prostate cancer: from a successful hormonal management to a review of the literature

**DOI:** 10.2144/fsoa-2021-0009

**Published:** 2021-04-30

**Authors:** Emilie Delchambre, Stéphane Rysselinck, Géraldine Pairet, Caterina Confente, Emmanuel Seront

**Affiliations:** 1 Department of Urology, Hôpital de Jolimont, Haine Saint-Paul,7100, Belgium; 2 Department of Pathology, Hôpital de Jolimont, Haine Saint-Paul, 7100, Belgium; 3 Department of Medical Oncology, Hôpital de Jolimont, Haine Saint-Paul, 7100, Belgium

**Keywords:** case report, hormone therapy, peritoneal carcinomatosis, prostate cancer, prostate-specific membrane antigen and PET

## Abstract

Metastases from prostate cancer involve mainly the bone compartment. However, visceral metastases are found in up to 49% of metastatic patients, occurring mainly in late stages of the disease, and are correlated with poor outcome. Peritoneal carcinomatosis is rarely described in literature, particularly when not associated with other distant metastatic lesions. We present the management of a patient with prostate cancer progressing on androgen deprivation therapy with description of omental involvement on ^68^Ga PSMA-PET. There was no ascite or other distant lesion, reflecting thus a specific tropism of the cancer in this patient who had no history of prostate surgery. Abiraterone acetate resulted in a long-lasting complete response. We also present a review focusing on this entity.

## Lay abstract

We report an atypical presentation of metastases from prostate cancer in a patient previously treated with radiotherapy and androgen deprivation therapy. Resurgence occurred as peritoneal carcinomatosis that was diagnosed at an early stage with ^68^Ga PSMA-PET. Abiraterone acetate resulted in long-lasting complete response in this patient. This case highlights the role of modern imaging in detecting early stages of complicating metastases, and to our knowledge is the first time a successful long-term response with abiraterone acetate has been observed in this entity.

Prostate cancer (PC) is the most frequently diagnosed malignancy and the second cancer-leading cause of death among men worldwide [1]. Even if localized PC is associated with an excellent outcome following radical prostatectomy or radiotherapy, advanced cancer with metastatic lesions remains a challenge for clinicians. PC metastases can invade any organ but they predominantly affect bone compartment, which provides a matrix rich in factors that stimulate the growth of tumor cells; approximately 90% of men with metastatic PC develop bone metastases during the cancer course.

Visceral metastases are not rare in PC. Pezaro *et al.* found that the incidence of visceral metastases on computed tomography (CT) scan at 9–12 months, 6–9 months, 3–6 months and within 3 months prior to death was 14, 22, 32 and 49%, respectively, suggesting that most patients developed visceral metastases late in the course of disease. This could reflect the natural history of the disease that is significantly improved with the current therapeutic strategies. The median intervals from cancer diagnosis or from castration resistant PC development to development of visceral disease were 4.6 and 1.6 years, respectively [2–4].

It is widely accepted that visceral metastases are a marker for poor prognosis in PC, independently of the treatment assigned [2]. The most common sites are lung followed by liver, pleura and adrenals [3]. Peritoneal involvement by PC cells is rarely described in literature, particularly when not associated with other metastatic location or ascites [5]. Rapoport reviewed the autopsy of 523 PC cases and found that only 13 cases had peritoneal deposits [6]. This frequency is probably underestimated in clinical practice due to the poor sensitivity of conventional imaging such as abdominal CT to detect early stages of omental implants [7,8].

The mechanism of this metastatic evolution is not clearly understood, even if some authors proposed iatrogenic spread following prostate surgery. Many questions remain unanswered concerning the prognosis of this entity as well as its optimal management. We report the case of a patient with PC and isolated peritoneal carcinomatosis, without bone or lymph node metastases and no history of previous surgery. Abiraterone acetate resulted in successful long-lasting control of the cancer.

## Case presentation

**Figure 1.**
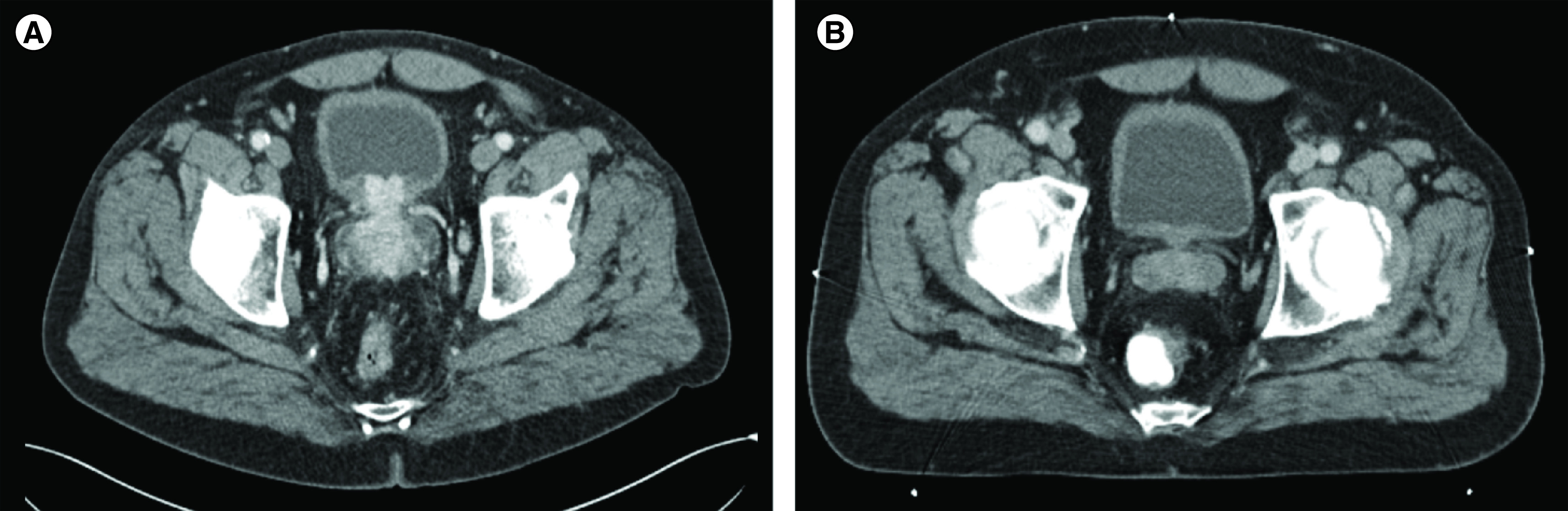
Radiological evolution on androgen deprivation therapy. **(A)** Initial abdominal CT showing locally advanced prostate cancer with bladder wall infiltration. **(B)** Abdominal CT after 12 months of androgen deprivation therapy, with a regression of the tumor and of the bladder infiltration. No peritoneal involvement was detected on the two CTs. CT: Computed tomography.

**Figure 2.**
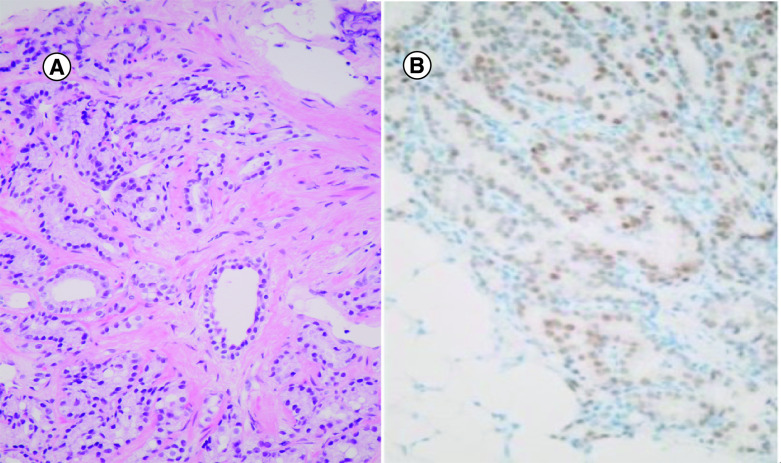
(A) Histological diagnosis of prostate cancer adenocarcinoma Gleason 7 (4 + 3) on primary prostate cancer. **(B)** Prostate adenocarcinoma on peritoneal biopsy.

**Figure 3.**
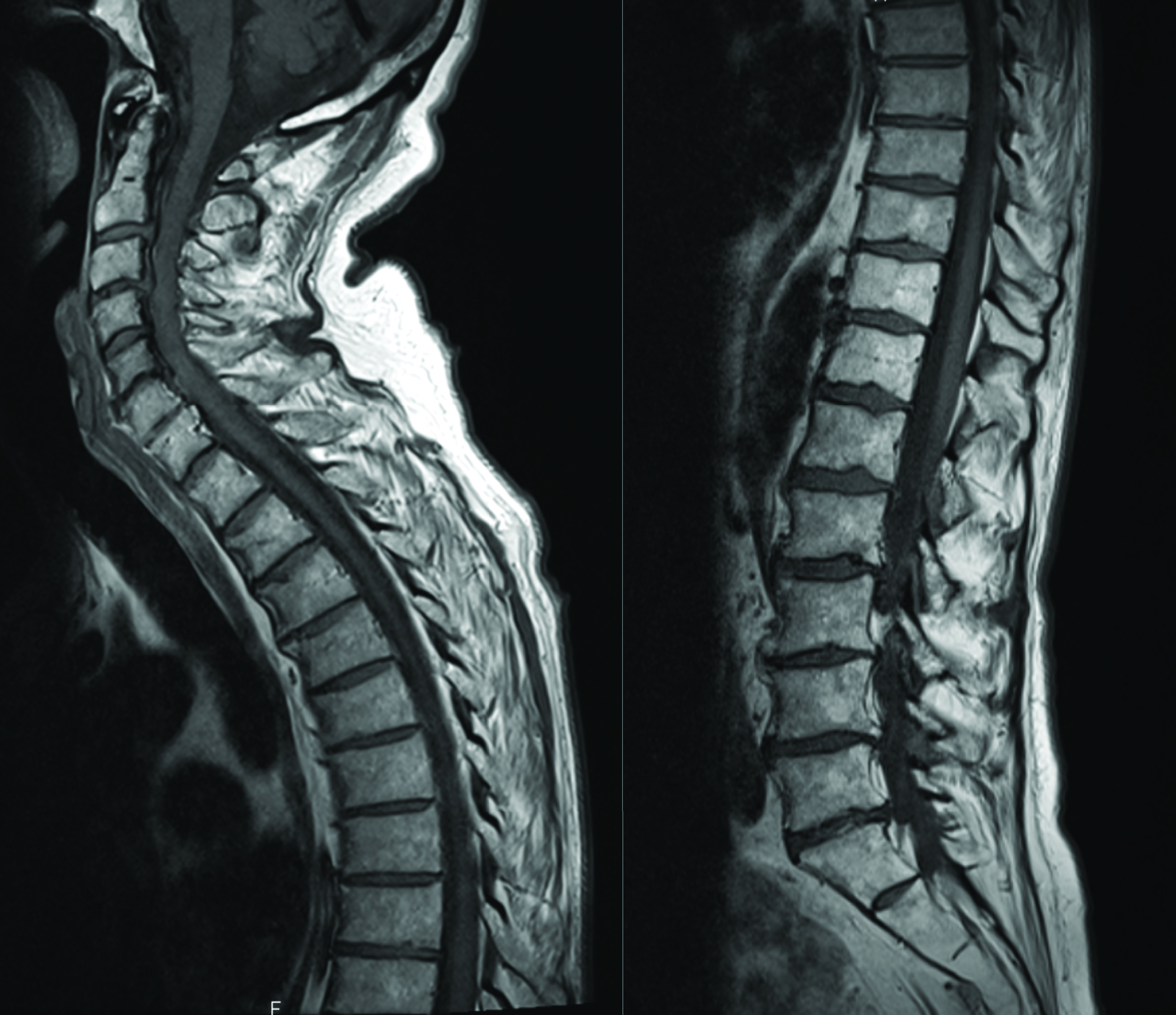
Axial skeletal MRI at cancer diagnosis showing no bone lesion.

**Figure 4.**
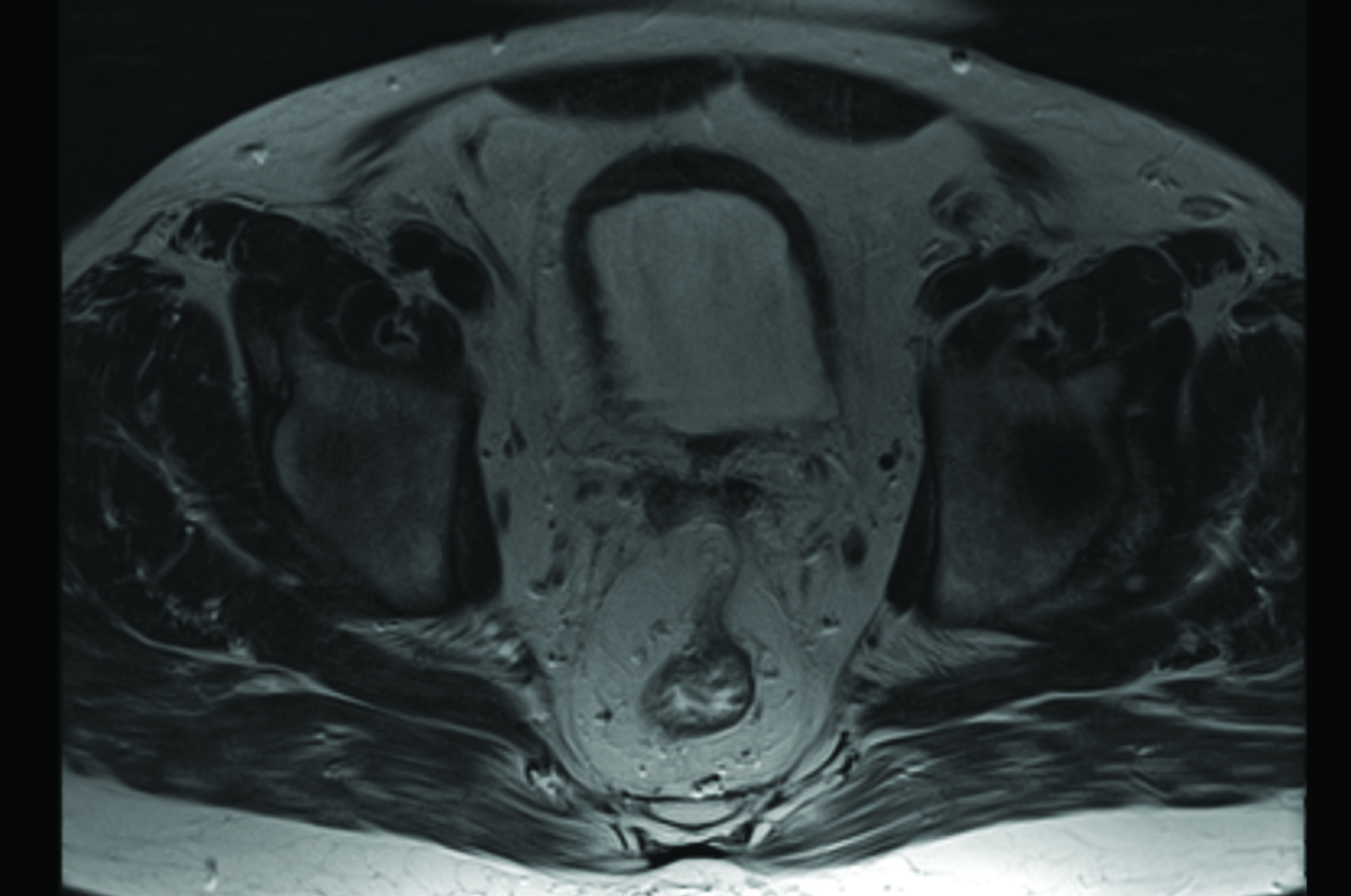
Pelvic MRI, performed after 12 months of androgen deprivation therapy, showing no suspect lymph node.

In 2009, a 64-year-old man was diagnosed with a PC based on prostate specific antigen (PSA) increase (117 ng/l). He had no relevant medical history except a tobacco-related obstructive broncho-pneumopathy; he did not describe any surgical history. Abdominal CT confirmed locally advanced PC with seminal vesicles and bladder invasion but without any evidence of lymph node or intra-abdominal anomaly (Figure 1A). Biopsy showed a moderately differentiated Gleason 7 (4 + 3) score with perineural infiltration (Figure 2A). Bone scan, thoracic CT and axial skeletal MRI did not show any secondary lesion (Figure 3). No pelvic MRI was performed at this time due to the fact that bladder invasion was clearly demonstrated on CT scan and that local treatment was not proposed first. The tumor was thus classified as T4 N0 M0 based on these imaging modalities. Due to the high value of PSA and the high risk of occult metastatic lesions, we first started androgen deprivation therapy (ADT) (leuproreline) that rapidly decreased PSA value after 1 month (25 ng/l); PSA continued to decrease and reached a value of 7 ng/l after 12 months. At this time, the thoraco-abdominal CT showed a regression of the primary tumor and of the bladder infiltration (Figure 1B). The pelvic MRI further did not show any any suspect lymph node (Figure 4); bone scan did not demonstrate any suspect distant lesion, as well as axial skeletal MRI. Radiotherapy (78 Gy on prostate area including the bladder infiltration location) was then performed; lymph nodes were also involved in the irradiated area (46 Gy) due to the high PSA level and the important local infiltration. This treatment, combined with ADT, resulted in PSA normalization (<0.2 ng/l) 6 months later. Leuproreline was stopped in March 2012.

**Figure 5.**
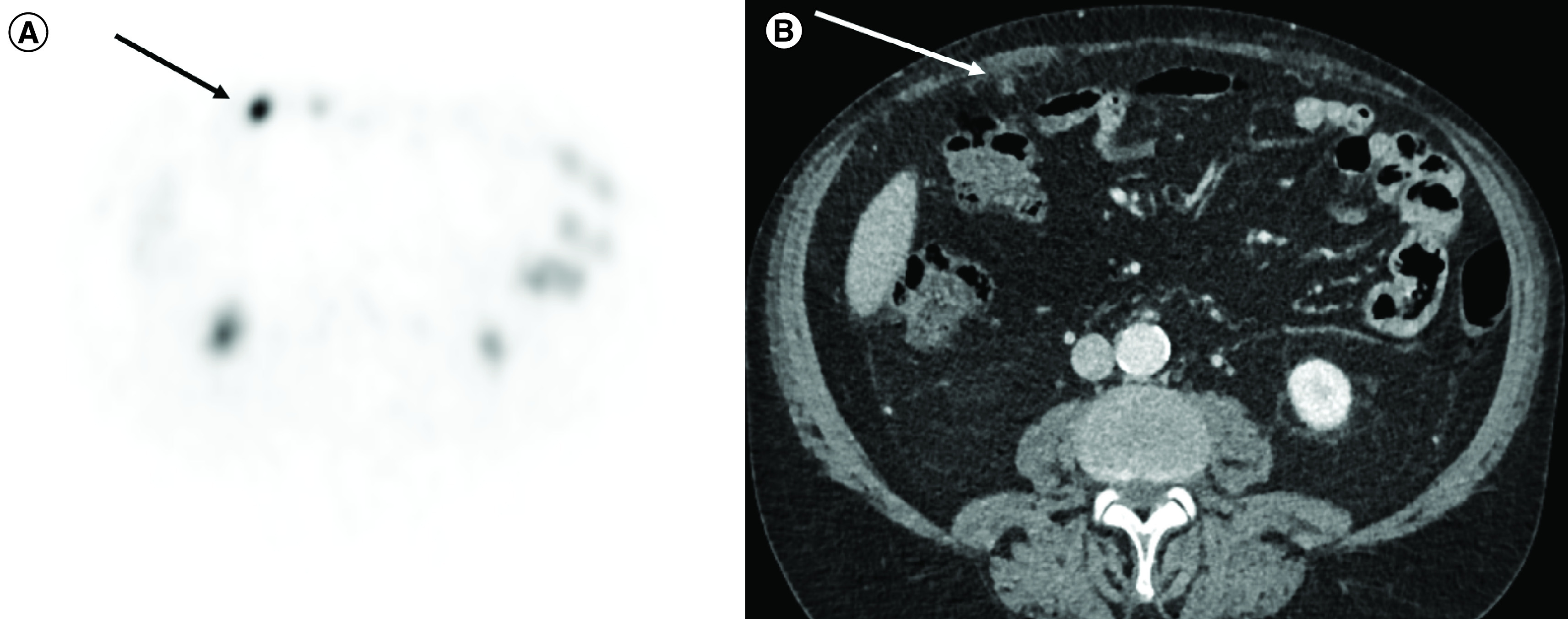
(A) PSMA-PET showing peritoneal implant (arrow). **(B)** Abdominal computed tomography that was performed on the same time and that initially misdiagnosed these peritoneal implants due to its very small size (arrow).

In 2014, PSA level raised progressively to 2.2 ng/ml with a PSA doubling time of 3 months. Bone scan and thoraco-abdominal CT did not reveal any suspect lesions; no modern imaging such as ^68^Ga PSMA-PET or whole-body MRI was done as it was not easily available. The very short PSA doubling time led us to start ADT (degarelix) that resulted in a rapid normalization of PSA after 3 months (<0.2 ng/l). PSA remained unchanged for 2 years but in 2016, it showed a rapid increase to 12 ng/l, reflecting development of castration resistance. Bone scan and thoraco-abdominal CT were considered as normal but ^68^Ga PSMA-PET showed five peritoneal infra-centimetric lesions without any other suspect lesions in bone or in lymph node. Based on ^68^Ga PSMA-PET imaging, peritoneal implants were retrospectively visualized on the synchronous abdominal CT (Figure 5) but not on the previous ones including the abdominal CT of the diagnosis. In order to exclude false positive lesion on ^68^Ga PSMA-PET, we decided to perform laparoscopic exploration that showed multiple suspect lesions in the peritoneal cavity; histology confirmed prostate adenocarcinoma without neuroendocrine, mucinous or small cell differentiation (Figure 2B). Abiraterone acetate (1000 mg daily + prednisone 10 mg daily) was initiated and PSA level rapidly decreased to 0.4 ng/l in 3 months and to 0.01 ng/l after 6 months. Treatment was well tolerated without any toxicity. Peritoneal lesions disappeared on abdominal CT. Three years later the patient remains in radiological complete remission and with undetectable PSA.

## Discussion

Peritoneal carcinomatosis is very rarely described in PC, particularly when isolated and not associated with other distant lesions, reflecting a potential specific way of dissemination and/or a specific tropism.

In this patient, peritoneal carcinomatosis was isolated; bone and other extra-abdominal synchronous macro-metastases were excluded by combining conventional imaging (bone scan and thoraco-abdominal CT) and modern imaging (^68^Ga PSMA-PET) suggesting that peritoneal cavity was the preferential and first homing in this patient. The mechanism of dissemination remains of course unknown. Some authors postulated iatrogenic spread following laparoscopic surgery and port-site metastases (Table 1). Our patient did not have previous prostate surgery, suggesting lymphatic or hematological dissemination. Although the primary tumor was locally advanced and invaded bladder wall, there was no direct peritoneal invasion visualized on first abdominal CT, on the 12-month pelvic MRI, on the ^68^Ga PSMA-PET and at laparoscopy. Few cases report similarly isolated peritoneal carcinomatosis occurring in patient without history of prostate surgery (Table 1); among the 13 patients with available data, 9 patients had a Gleason score >7 at initial cancer histology, including one patient with neuroendocrine differentiation, suggesting that these aggressive variants could be associated with development of omental involvement.

**Table 1.** Review of peritoneal carcinomatosis associated with prostate cancer.

Age (years), ethnicity	Initial histology of PC	Initial treatment	Interval between PC and Mets	CRPC at diagnosis	Ascite	Distant metastasis	Diagnosis modalities: PSA; imaging biopsy	Treatment	Outcome/ comment	Ref.
**Isolated peritoneal carcinomatosis without previous history of abdominal surgery**										
74	Adenoc Gl. NA	Docetaxel + AA	NA	Yes	No	No	PSA 200 ng/l; PSMA-PET; CT-guided biopsy	Cabazitaxel	OS = 10 months	[9]
59, African	Adenoc Gl. 7	None	0	No	Yes	No	PSA 54.6 ng/l; Incidental finding during hernia surgery	ADT	OS = 13 months	[10]
75	Adenoc Gl. 9	ADT	3 years	Yes	Yes	LN	PSA 10.3 ng/l; Abdominal CT; CT-guided biopsy	ADT	OS = 4 months	[11]
75	Adenoc Gl. 9	ADT	6 years	Yes	Yes	No	PSA 74 ng/l; Abdominal CT; Paracentesis	Docetaxel	Regression of ascite; Alive at 18 months; OS = NA	[12]
75	Adenoc Gl. 9 cT3	None	0	No	No	LN	PSA 42 ng/l; Incidental finding during LND	ADT	Biological and radiological response; PFS = 14 months; OS = NA	[13]
58, African	Adenoc Gl. NA	None	0	No	Yes	No	PSA NA; Abdominal CT; Peritoneal biopsies (laparotomy)	RT + ADT	Radiological response; Alive at 6 months; OS = NA	[14]
70, Caucasian	Adenoc Gl. 9	RT + ADT	4 years	Yes	Yes	No	PSA 262 ng/l; Abdominal CT; Paracentesis	Thalidomide	No response; OS = NA	[15]
76, Caucasian	Adenoc Gl. 6	TURP	4 years	No	Yes	No	PSA 364 ng/l; Abdominal CT; Paracentesis	Orchidectomy	Biological response; Alive at 18 months; OS = NA	[16]
67, Indian	Adenoc Gl. 8	TURP + ADT	2 years	Yes	Yes	No	PSA 82 ng/l; Abdominal MRI; US-guided biopsy	Docetaxel	Clinical response after 2 cycles of docetaxel; OS = NA	[17]
65	Adenoc Gl. 7	ADT + ketoconazol	9 years	Yes	Yes	No	PSA 27 ng/l; Abdominal CT; CT-guided biopsy	Docetaxel	OS = NA	[18]
63	Adenoc Gl. 8 and neuroendocrine cT3NxMx	RT + ADT + estramustine	12 years	Yes	Yes	No	PSA 1 ng/l; Abdominal CT; Paracentesis	Docetaxel	OS = 33 months	[19]
91	Adenoc Gl. NA	None	0	No	Yes	No	PSA 19.7 ng/l; Abdominal CT; CT-guided biopsies	Palliative care	OS = NA	[20]
60	Adenoc Gl. 8	RT + ADT	3 years	No	Yes	No	PSA 330 ng/l; FDG PET/CT; Paracentesis	NA	OS = NA	[21]
68	Adenoc Gl. 9	ADT	1 years	Yes	Yes	Rectum	PSA 79 ng/l; Abdominal CT; Paracentesis	ADT + IFN-α	OS = 4 months	[22]
73	Adenoc Gl. NA	RT	9 years	No	Yes	LN	PSA 9 ng/l; Abdominal CT; Peritoneal biopsies (laparotomy)	Palliative care	OS = 3 weeks	[23]
70, Indian	Adenoc Gl. 7	PRT (aborted)	1 week after prostatectomy	No	Yes	No	PSA 38.2 ng/l; Abdominal CT; Paracentesis	ADT Docetaxel Cabazitaxel	PFS on ADT = 5 months; PFS on docetaxel = 6 months; PFS on cabazitaxel = 4 months; OS = NA	[24]
62	Adenoc Gl. 9 cT2cN0M0	Aborted RALP + LND	0	No	No	No	PSA 13.3 ng/l; Incidental finding during LND; Peritoneal biopsies (laparoscopy)	ADT	Biological response; OS = NA	[25]
**Isolated peritoneal carcinomatosis associated with previous history of abdominal surgery**										
57	Adenoc Gl. 7 pT3bN1	RALP Salvage RT + ADT	13 years	Yes	No	LN	PSA 15.7 ng/l; Incidental finding during hernia repair	Enzalutamide	Iatrogenic spreading proposed by authors; OS = NA; PSA nadir 8 months after onset of enzalutamide	[26]
60	Adenoc Gl. 8 pT2cpN0R0	RALP Adjuvant RT + ADT	11.5 years	No	No	No	PSA 30.6 ng/l; PSMA-PET-MRI; CT-guided biopsy	ADT + Docetaxel	Iatrogenic spreading proposed by authors; OS = NA	[26]
58	Adenoc Gl. 7 pT3bpN0	RALP + LND Salvage RT	3 years	No	NA	No	PSA 4.3 ng/l; Choline PET-CT; CT-guided biopsy	RT on port site metastasis	Iatrogenic spreading proposed by authors; OS = 6 months	[27]
60	Adenoc Gl. 8 pT3apN0	RALP + LND Salvage RT	7.5 years	No	NA	No	PSA 1.9 ng/l; Choline PET-CT; CT-guided biopsy	Cryoablation of port site metastasis	Iatrogenic spreading proposed by authors; OS = 6 months	[27]
59	Adenoc Gl. 7 pT2apN0	RALP + LND Salvage RT + ADT	5 years	No	NA	LN	PSA 1.5 ng/l; Choline PET CT + MRI; Guided biopsy	ADT + docetaxael	Iatrogenic spreading proposed by authors; RT on LN with no sign of recurrence	[27]
62	Adenoc Gl. 7 pT2cpN0	RALP + LND	2 years	No	NA	No	PSA 1.5 ng/l; Abdominal CT; Guided biopsy	Omentectomy + LND	Iatrogenic spreading proposed by authors; PSA recurrence at 2 years, undectectable after starting ADT	[27]
59	Adenoc Gl. 8 pT3bpNx	RALP Salvage RT	4 years	No	NA	LN	PSA 5.1 ng/l; Intra-operative finding; Surgery	Peritoneal metastasectomy + LND + docetaxel + ADT	Iatrogenic spreading proposed by authors; OS = 60 months at least	[27]
55	Adenoc Gl. NA pT3apN0	RALP + LND salvage RT + ADT	3 years	No	NA	No	PSA 2.8 ng/l; Choline PET CT + MRI; Surgery	ADT + docetaxel	Iatrogenic spreading proposed by authors; OS = 34 months	[27]
69, Japanese	Adenoc Gl. 8 pT3a	LRP + RT + ADT	9 years	No	Yes	No	PSA 168 ng/l; Abdominal CT; Autopsy	None	Iatrogenic spreading; proposed by authors; OS = 6 months	[28]
90	Adenoc Gl. NA	Open PRT + RT	10 years	Yes	Yes	No	PSA 780 ng/l; Abdominal US; Paracentesis	Palliative	OS = 3 months	[29]
65	Adenoc Gl. 9 and neuroendocrine cT4NxMx	LRP Salvage RT + ADT	9 years	Yes	No	No	PSA 14 ng/l; Abdominal CT; No biopsy	Docetaxel	Iatrogenic spreading proposed by authors; OS = 21 months	[19]
50	Adenoc Gl. 7 pT4NxMx	LRP Adjuvant RT + ADT	3 years	Yes	Yes	No	PSA NA ng/l; Abdominal CT; Parencentesis	Docetaxel	Iatrogenic spreading proposed by authors; OS = 36 months	[19]
74, Caucasian	Adenoc Gl. 7	RALP + LND Salvage RT + ADT	14 years	Yes	No	No	PSA 40.5 ng/l; Abdominal CT; CT-guided biopsy	Docetaxel	Iatrogenic spreading proposed by authors; SD at 5 month; OS = NA	[30]
60	Adenoc Gl. 7	RALP Adjuvant RT-ADT	30 months	Yes	No	No	PSA 5.6 ng/l; Abdomnial CT; CT-guided biopsy	Total omentectomy + AA	Iatrogenic spreading proposed by authors; Radiological and biological response; OS = NA	[31]
65	Adenoc Gl. 7 pT2c	RALP + ADT	10 years	Yes	No	No	PSA 6.6 ng/l; Abdominal CT; Laparoscopy	AA	Iatrogenic spreading proposed by authors; Radiological and biological response; OS = NA	[32]
65	Adenoc Gl. 9 pT3b	RALP + LND Adjuvant RT + ADT	2.5 years	No	Yes	No	PSA 93 ng/l; Abdominal CT; Paracentesis	Docetaxel; Mitoxantrone; Cabazitaxel; Palliative care	Iatrogenic spreading proposed by authors; OS = NA	[32]
77	Adenoc Gl. 9 pT3a	LRP + LND Adjuvant ADT	2 years	Yes	No	No	PSA 0.67 ng/l; Abdominal MRI + FDG PET; Surgery	Surgery; AA; Chemotherapy	Iatrogenic spreading proposed by authors; Response during 6 months; OS = NA	[33]
70, Caucasian	Adenoc Gl. 8	Radical PRT + ADT	7 years	Yes	Yes	No	PSA 407 ng/l; Abdominal CT; No biopsy	Chemotherapy	Radiological and biological response; Alive at 5 years	[34]

AA: Abiraterone acetate; Adenoc: Adenocarcinoma; ADT: Androgen deprivation therapy; CRPC: Castration-resistant prostate cancer; CT: Computed tomography; Gl: Gleason; LN: Lymph node; LND: Lymph node dissection; LRP: Laparoscopic radical prostatectomy; NA: Not available; OS: Overall survival; PC: Prostate cancer; PCa: Prostate cancer; PRT: Prostatectomy radical total; PSA: Prostate specific antigen; RALP: Robotic-assisted laparoscipic prostectomy; RT: Radiotherapy; SD: Stable disease; TURP: Transurethral resection of prostate; US: Ultrasound.

Peritoneal carcinomatosis was diagnosed at a very early stage with ^68^Ga PSMA-PET, before the apparition of ascites or symptoms. Conventional imaging such as CT initially misdiagnosed peritoneal involvement and could thus in clinical practice result in delay in diagnosis, explaining the low frequency of nonascitic peritoneal carcinomatosis reported in literature. When analyzing the patients from Table 1, among the ten nonascitic patients, four patients were diagnosed with modern imaging (^68^Ga PSMA-PET, PET-Choline or MRI) [9,26,27,33] and four were diagnosed incidentally during surgery [10,13,25,26]. The increasing use of modern imaging such as ^68^Ga PSMA-PET will probably allow more frequent atypical metastatic localization at early stage of development.

It remains unknown whether omental carcinomatosis should be considered as a poor prognosis factor. The majority of cases were treated with ADT ± docetaxel with, in some patients, biological and radiological response. Only four patients have been treated with new generation hormonal agents (three with abiraterone acetate and one with enzalutamide) with description of response without any precision in outcome [26,31–33]. In our patient, due to the prior sensitivity to ADT (biological response lasting more than 1 year), we started abiraterone acetate that resulted in rapid decrease of PSA and long lasting complete radiological response for more than 4 years.

Limitation in our interpretation includes the absence of pelvic MRI and ^68^Ga PSMA-PET at diagnosis and at biochemical recurrence (unavailable in current practice in 2009 and 2014). We cannot exclude presence of metastases (bone and/or omental) that could have initially regressed with ADT and led to emergent clones with particular tropism for omental compartment. Whether a delayed diagnosis of this carcinomatosis would had led to similar outcome remains also unknown; however, this case highlights the role of modern imaging in detecting early stage of such potentially complicating metastases. It also opens discussion concerning potential and unknown dissemination ways and specific tropism of PC cells.

To our knowledge, we are the first to demonstrate a such successful long-term response with abiraterone acetate in this entity.

## Conclusion

Peritoneal carcinomatosis is rarely described in PC, particularly when not associated with other metastatic lesions. In this case, we describe the role of ^68^Ga PSMA-PET in allowing detection of peritoneal metastases at early stage and the successful response to abiraterone acetate.

## Future perspectives

The management of PC is significantly improving with new drugs and new strategies. The better understanding of the PC pathogenesis helps to develop new targeted therapies, increasing the treatment options and offering tailored strategies. New imaging modalities such as metabolic imaging also allow better stratification of cancer, which can improve early management of patients. Improvement in genomics will also help to predict patients at high risk of resurgence in order to improve their follow-up.

**Executive summary**

- Visceral metastases are frequently observed in prostate cancer (PC), mainly in late stages of the disease.
- Atypical presentation such as peritoneal involvement can occur in PC and could be isolated. This peritoneal involvement can occur regardless of previous surgery on PC. However, some authors have presented case series highlighting the potential relationship with PC surgery.
- Modern imaging approaches such as ^68^Ga PSMA-PET allow early and accurate stratification of PC.
- Abiraterone acetate is a new hormonal agent improving the outcome of patients with metastatic PC. Abiraterone acetate was shown to be efficient in peritoneal carcinomatosis from PC, resulting in long-lasting complete response.
- Even in the case of initially locally advanced cancer, radical treatment should be offered to any patient, as prognosis is drastically improved with new therapeutic strategies and in order to prevent local complications.
